# Supplementary material for: Acceptance and compliance with micronutrient powder (MNP) among children aged 6–23 months in northern Nigeria
Source: PLOS Glob Public Health. 2022 Oct 17;2(10):e0000961. doi: 10.1371/journal.pgph.0000961 (PMC10022258; doi:10.1371/journal.pgph.0000961)
Supplement: S1 Dataset — (ZIP) [file pgph.0000961.s008.zip › S8_Dataset/In-depth Interviews.zip!/In-depth Interviews/080715_A_GA_AS_2_CG.pdf]

AN INDEPT INTERVIEW CONDUCTED WITH A CAREGIVER IN GWANDOKI COMMUNITY IN GANYE LOCAL GOVERNTMENT AREA OF ADAMAWA STATE.

I: Please descibe for me the people in your immediate family living in your household.

R: There are ten adults and three young children living in this household.

I: Please tell me the gender and the age of your child.

R: As you can clearly see, my child is a female and is 21months old.

I: Your earlier mentioned young children living in your household, please tell me about them.

R: Yes there are 2 young children in this household plus my child making a total number of 3 young children. The first child is my husband's niece and she is 18months, while the other child is a son of one of our relation and he is 12months old.

I: we have heard of food challenges for young children in this setting related to the seasons. Please tell me about it.

R: There are no food challenges for young children in this household. We use guinea corn and mostly maize to feed our young children and these foods are always available season in and season out. Sometimes we use mashed groundnut to make kunu for our young children and this groundnuts are always available even it is not the season for it, my husband gives me money to buy such foods.

I: I've heard in other interviews that caregivers often give their children 6-23months TEA. Could you please explain tea.

R: Of a truth, we have never poured or mixed the product into tea. We were told to mix the product into semi solid food like kunu and the kunu should be fetched in a small cup exactly the quantity that my child can eat all of it. That is exactly what we have been doing since we were given the packets of the product.

I: Please explain to me what tea is.

R: Most people particularly in this community make their tea with lipton teabag boiled in hot water and a little sugar is added into it. But here in this household, we make our tea with hot water and we add a little milk and milo into it with a little sugar also.

I: Now i want us to talk about the MNP that was given to you some time ago, please tell me about your experience with the product.

R: We were given this product some time ago and we were told that the product is meant for young children 6- 23months. My experience with this product has been going smoothly, there hasn't been a day that i had difficulty in using this product. The food i use in mixing the product has been readily available since when i was given the product to use, my husband has been very supportive because he stores the product himself. My experience with this product is exciting to me and i am very grateful to the federal ministry of health for introducing this product to us.

I: Okay let's talk about the positive aspects of your experience with this product.

R: This product has made my child to be very strong and healthy, the product has boost the appetite of my child, she eats very well and she eats almost everything. When we started giving our child this product, her eating pattern changed, she eats too much and the way she sucks my breast has also increased, i was telling the father that i would soon stop breastfeeding her because she wouldn't allow me rest and the father insists she continues. As you can see, since you came i have been breastfeeding her right to this moment she has not rested. Before now, she may suck my breast for just 5 minutes or there about but now, she can go for an hour none stop and that makes me hungry and tired.

I: I would like you to please discuss the benefits of this MNP to your child.

R: This product has made my child to be full of strength and vitality, she eats all of her kunu and any other food given to her, she is not picky but eats everything given to her. Her appetite has increased a great deal. This product has made my child to be full of activity, she plays a lot now unlike before that she would just sit quietly. She cries a whole lot too, everything about her has changed. She sleeps a lot also, in the evening when she starts sleeping by 7pm, she does not wake up until the next morning and she does not wake up at mid night to suck breast milk.

I: Please tell me some stories related to the improvement in the health of your child compared to before using this product.

R: Aside from the fact that my child is so full of strength now, i have noticed that my child is now very intelligent compared to before using the product. Before now if i send her to help me pick something, she would just stare at me and wouldn't understand what i am talking about but now, if i send her to the room or anywhere else she would go directly to that thing i sent her to get and return with it. Anything you give to her now, she would collect it right away and eat it, give her orange she would take it, give her coconut or banana, she would eat up everything.

I: Okay Please describe to me the challenges you had during this few weeks that you started using this product.

R: There hasn't been any challenge at all, we have the semi solid food to use alongside the product and the child is very cooperative. Everything is going normal.

I: Please tell me about some barriers that made it difficult to use this product appropriately

R: At first when we started using the product, we noticed that the child had fever or running temperature, i was a little scared and wanted to discontinue using the product but my husband told me to still continue, after some time the fever stopped and my child's condition improved.

I: Some caregivers mentioned that it was hard to store the MNP without it being taken by others or eaten by rodents. Please tell me if you have a similar experience.

R: In this household my husband instructed that the product should be stored and locked up in a travelling box, our travelling boxes here are made from metal and rodents can't have access to the product. Every day i pick 1 sachet of the MNP and lock up the box immediately. We are lucky that in our room there is no such thing as rodents.

I: That's very good. Now please tell me about the side effect of this product on the child.

R: There has been one or two side effects my husband and i have noticed in our daughter. When my child started using this product, we noticed she had some heat rashes all over her body and there were also some boils on her head, she had diarrhea and fever also. My husband told me to continue to give my child the MNP and not skip a day. After some days, the heat rashes disappeared and the boil on her head dried up too, the diaahea seized also.

I: You just mentioned that your child had some side effects like diarrhea, heat rash and boil on the head. Please tell me how you handled all that.

R: We continued to use the MNP for the child but my husband bought some antibiotics for the rash and for the boils, he also bought vitamin C syrup for our child. Later on the diarrhea and the fever all seized just like that.

I: Some mothers find it difficult to remember to use this product on a daily basis. I would like you to tell me if you have a similar experience.

R: The mother that forgets to use this product on daily basis is a careless mother, how could you call yourself a mother and forget something of great importance to your child's health. I do not forget to give the product daily to my child. Besides, you just counted the sachet in the packet, did you find any fault in it?

I: No i didn't.

R: Exactly what i am telling you. Everyday my husband ensures that i make kunu and mix the MNP in it and give our child all of it before he goes to the farm. That is what he does daily and there hasn't been a day that we forgot to give this child this product.

I: Let’s talk about the child’s feeding and response to the MNP. Please tell me how you feed the MNP to the child usually.

R: Every day after making my kunu, i fetch a little in a small cup like 4-5 spoons. I allow it to get cold, not completely cold but a little warm. I pick one sachet of the product and mix it into the kunu, i would mix and mix with a small spoon until it has blended into the kunu very well after which i will call or carry my daughter and feed her using that small cup and spoon.

I: Please tell me about the food you mix the product with.

R: Mostly in this community, we feed our children with either guinea corn flour or millet flour cooked together with mashed groundnut and hot water. Ever since, i started giving my child this product, i particularly use the guinea corn flour and mashed groundnut and i add a little sugar to taste because that is what my child is used to right from when i introduced her to complimentary breastfeeding at 6months.

I: Okay please tell me when the MNP was usually given to the child.

R: Like i told you earlier, my husband ensures that i give the MNP to our child before he leaves for the farm and farmers usually leave very early so, i give her the MNP everyday by 6am or 7am

I: I would like you to talk about the extent that your child likes or dislikes the taste of the MNP

R: My child does not reject the product at all, she likes the way it is, every morning when the dad brings out one sachet and keeps for us to use, i send her to go pick it and bring it, she would go and pick the sachet and bring it for me to mix into her kunu without any hesitation.

I: Please discuss how the MNP has impacted the current diet of your child.

R: This product has boost the appetite of my child because her appetite has increased a great deal, she eats almost everything given to her. Once i make her kunu in the mornings, she would eat all of it without remaining even a little and now, she consumes more breast milk and drinks a lot of water also like i earlier told you, she can go for an hour sucking breast milk without a break. Honestly i have noticed a great change in her diet and my only concern is the way she consumes my breast milk. It looks like she wants to suck all of it at once.

I: In other communities we noticed that sharing of the MNP occurred. Could you talk about any sharing of the MNP when you used it.

R: Since the day the product was given to us, we have never given out even one sachet out, our child is the only person that has been eating the product. We have not given the product to even our next door neighbour who may have children of the same age with our daughter, all i do is to tell them to wait for their product to be given to them because if i say i would give them part of it, it would not benefit their child and my child would have limited dose and that is what i can afford to do.

I: You earlier mentioned some young children who are between 18months and 12months living in this household. Please tell me if you give them some of this product to eat also.

R: No I don’t give them part of it to eat. We were instructed at the health centre to not share this product or give part of it to our neighbours, when we all summoned and it was clearly said that we shouldn’t take part of it and give anyone else, we should recommend them to go to the health centre also. I know that if i don’t give my daughter all of the 2packets given to me, she wouldn’t get the required benefits of the full doze of the product, so why should i even consider sharing it.

I: I would like to know whether you share this product due to food shortage.

R: Like i clearly told you, i have never shared this product due to any reason, it is strictly meant for my daughter and that is exactly what i do.

I: Please tell me whether you or the father has eaten the product too.

R: That’s very funny, how can myself or the dad take part of it to eat. Would it benefit us in any way, anything that is given for children would not impact on the health status of adults and we are concerned about the health of our child so why should we eat part of it.

I: I would like to know if the leftovers of the MNP are eaten by other young children in your household

R: No my child eats all of it and there hasn’t been any leftovers since we started given her the MNP, as a matter of fact, we were instructed to fetch a little portion of the kunu and mix the MNP, the portion i fetch is usually too small to be left uneaten.

I: Please discuss whether 60 sachets will be enough for 60days.

R: Yes 60satchet would be enough for 60days.

I: Please tell me why.

R: Because each packet contains 30 sachets making a total of 60satchets to be used one daily for 60 days, if i take one sachet daily for 60days, it would be enough for me to use in 60days exactly. Just like now, i have used 19 sachets for 19 days, i still have 41 sachets to use for the remaining 41 days and that makes a total of 60days.

I: That’s impressive. Now that you have used the MNP, could you talk about how you perceive it.

R: This product is very good and has improved the health of our child in that, it has made her strong and fit, it has improved the way she eats, she plays with so much energy and does not get tired easily, she cries very loud with so much strength (laughing). I personally perceive the MNP as something with a number of important compositions that works wonders in children.

I: Please tell me whether you see the MNP as a medicine or a food or something else.

R: I see this product as a medicine, because the product produces results that only medicine can.

I: Please tell me more

R: Yes i see this product as a medicine because first and foremost, it was distributed by a health worker, any product distributed by a health worker is a medicine. That is my own understanding.

I: Could you please tell me whether you would purchase this product if it were cheaply on sale in your community.

R: Yes i would purchase the MNP without waste of time.

I: Please tell me why you said so.

R: Because i have seen the benefits of using this product on my child, i have seen the improvement in her health, i have seen an improvement in the way she eats, plays and cries. I have noticed an improvement on her intelligence and her weight has also increased because you cannot carry her with one arm now but with both arms. I am highly impressed by the tremendous change i see in her and i am ready to purchase it if it were to be sold.

I: Okay, i would like to know how much you would be willing to pay for each sachet.

R: I am willing to pay 50 naira per sachet making the total of 1500 naira per packet.

I: You are doing a great job, i am well pleased with the kind of responses i am getting from you. Now please tell me some ways that you believe would be effective for promoting this MNP to caregivers in this community so that they would comply with using it appropriately.

R: For people like us that have tasted the product on our children, we can promote the product by encouraging mothers to give this product daily to their children and they would record a huge difference in the health status of their children, the eating pattern, the way the child plays and the great display of energy by the child is interesting to be noted. We can tell them in our local language about the benefits of using the product for young children and urge them not to be left out of this great thing the federal government is doing.

I: I would like you to talk about the people that influence your opinions and knowledge about feeding practices.

R: Our husbands and our mother in laws are the people that mostly influence our knowledge and feeding practices. They are more knowledgeable and experienced than us. In my household, my husband gives instructions on what and how i should feed my child daily, he

tells me how to maintain a good hygiene in terms of the meal preparation and feeding practices of our child and i follow the instructions carefully since he is the head.

I: Please tell me who influences your decision regarding feeding your baby with MNP.

R: Like i rightly stated to you, my husband is the person that ensures that the MNP is given to our child daily before he leaves for the farm, he would sit and wait for me to make the kunu and wait for it to be cold a little and then bring one sachet to be mixed with the kunu and also wait until our child eats all of it. He was the one that brought the idea of where and how the MNP should be stored and he stores it himself. It is my husband that influences me to use the MNP daily and now, i am already used to the practice.

I: Could you please tell me where would be a good place to disseminate health information related to MNP.

R: The best place would be at the health centre exactly the way it was done before now when this MNP trail began, we were all summoned at the clinic and we were told how, when and with what we should use the product. I still prefer that things should be done like the way it was done previously.

I: Please tell me why you said you prefer to receive health information related to MNP at the clinic.

R: Because at the health centres, the topics are explained to us carefully in our local language (chomba) and we gain an easier understanding when we are all gathered together, we can ask questions for more clarifications and our questions are well attended to. That is why now i know how the product can be used and i also know that it should not be shared.

I: Could you please tell me who in your community you trust the most for receiving messages about infant and young child feeding.

R: We trust our ward head the most, because each time there is anything relating to the improvements of children’s health, he goes from house to house to instruct us on how to carry out such practices. We believe so much in him because he is concerned about the general welfare of children living in this community.

I: I would like you to tell me what forms of media you prefer for receiving messages about infant and young child feeding. Do you prefer brochure/flyer/poster or word of mouth, radio, sms or any other means.

R: I prefer the word of mouth.

I: Please tell me from whom would you prefer to receive the word of mouth from.

R: I would like the health workers to tell me the massages of the MNP or any other health issue why because i understand them easily and i am already used to hearing health related

messages from them and i also get the opportunity to ask some questions about women’s health and stuffs like that.

I: I would like to know your preferred way of receiving education regarding IYCF and MNP.

R: It is through that same medium i just told you about, i would like to receive such messages or any other health related message from the health workers either from word of mouth or from the health workers at the clinic, any one is fine by me.

I: Please tell me about any successful health education campaign in this community.

R: Before now there has been health education on immunization, we were all summoned and we were instructed that when the health workers come for routine immunization, we should comply and bring our children from the rooms so that they can be immunized. That campaign was a huge success and people came out in large numbers.

I: Please tell me what made it a huge success as you earlier mentioned.

R: What made it a huge success was because parents and mostly caregivers are so interested in the welfare and the improvement in the well being or health conditions of their children. Whenever we are told that the government is about to introduce or has introduced a programme for children, we turn out in mass because we as mothers would like to always see our children grow in a healthy manner.

I: Could you please tell me the best community channels to distribute the product.

R: Just like the way it was done previously, the best channel is house to house distribution of the MNP.

I: Please tell me why you prefer the house to house distribution.

R: Because that was the way it was done before now and i enjoyed it in that manner, if they are coming let’s say tomorrow, our ward head would go round and inform us before time so that, we wouldn’t go the farm on the distribution day. When they come for the second distribution, they should still repeat the same things they told us earlier on how to use the product and with what to use the product with, it is good that way.

I: I want to hear some specific messages you think would effectively promote the product.

R: caregivers should be told that this product is very good because it can make your child to become strong and healthy, it can make your child to play very well. They should be told that this product is very good for young children.

I: Please discuss some messages that should be used to explain MNP to caregivers.

R: The things i just mentioned should be told to them and explained to them. It should be explained to them that, the federal government has introduced a product for young children

and not a dime was demanded from us all they need is our co-operation and acceptance so that this product can be used for young children, so therefore they shouldn't be left out of this golden opportunity to improve the health conditions of their children. And this product was brought down to our door steps, it is only wise that we embrace it.

I: Could you please tell me some specific messages to ensure that it is used appropriately.

R: They should be told to use it daily and not store it in their rooms, they should be told that before they can record any difference in their young children, they should ensure not to skip a day, even when they forget to give the product to the child in the morning, they can give them in afternoons or in the evenings. When they are told these messages clearly, they would use this product appropriately.

I: I would like you to tell me some effective messages to promote its use but limit the sharing of it.

R: They should be told not to give even one sachet out to a friend or a loved one, if you love your neighbour or friend that much, you should refer her to where she can get the 2 packets and not remove any sachet from your child's personal use. They should be told that if you share it, you wouldn't see the full benefits of the product.

I: Finally is there anything else that you can share about your experience with this product.

R: All i can say now is to offer my heartfelt gratitude to you guys that brought this product to us, we sincerely appreciate it. There is nothing more i can say about my experience with the product that i haven't said earlier, all i can say now is thank you.

I: Please tell me anything else about the product, placement, price, promotion.

R: There's nothing more about the product at all, the product is like milk powder and my child likes it so, there is nothing to say more about it.

I: Lastly i would like you to recommend anything else to develop an effective program.

R: There is nothing else but that the federal government should bring more of this product so that every mother who has young children 6-23months can have access to it that way, it Can limit sharing of this product, because even in this household we have young children who fall between 6-23months that do not have the product, i know of several other caregivers who have young children 6-23months that have not been given this product also. Please the government should ensure that no household is left out in the distribution of the product.

I: Thank you so much for your time and response, you have done a great job.
